# Design and Experimental Evaluation of a Hierarchical LoRaMESH-Based Sensor Network with Wi-Fi HaLow Backhaul for Smart Agriculture

**DOI:** 10.3390/s26092645

**Published:** 2026-04-24

**Authors:** Cuong Chu Van, Anh Tran Tuan, Duan Luong Cong

**Affiliations:** Faculty of Electronics Engineering 1, Posts and Telecommunications Institute of Technology (PTIT), Hanoi 100000, Vietnam; cuongcv@ptit.edu.vn (C.C.V.); anhtt@ptit.edu.vn (A.T.T.)

**Keywords:** smart agriculture, internet of things (IoT), wireless sensor networks (WSN), Wi-Fi HaLow (IEEE 802.11ah), LoRaMESH, hierarchical network

## Abstract

Large-scale smart agriculture requires reliable and energy-efficient wireless connectivity to support distributed environmental sensing across wide rural areas. However, existing low-power wide-area network (LPWAN) technologies often face limitations in scalability, reliability, or infrastructure dependency when deployed in large agricultural fields. This study presents the design and experimental evaluation of a hierarchical sensor network architecture that integrates LoRaMESH for multi-hop sensing communication and Wi-Fi HaLow as a sub-GHz backhaul for data aggregation and cloud connectivity. In the proposed system, LoRaMESH forms intra-cluster sensor networks using a lightweight controlled flooding protocol, while Wi-Fi HaLow provides long-range IP-based connectivity between cluster gateways and a central access point. A real-world deployment covering approximately 2.5km×1km of agricultural area was implemented to evaluate the performance of the proposed architecture. Experimental results show that the LoRaMESH network achieves packet delivery ratios above 90% across one to three hops, with average end-to-end delays between 10.6 s and 13.3 s. The Wi-Fi HaLow backhaul demonstrates high reliability within short to medium distances, reaching 99.5% packet delivery ratio at 50 m and 89.68% at 200 m. Energy measurements further indicate that the sensor nodes consume only 21.19μA in sleep mode, enabling long-term battery-powered operation suitable for agricultural monitoring applications. These results indicate that the proposed hierarchical architecture is a feasible connectivity option for the tested large-scale agricultural sensing scenario. Because no side-by-side LoRaWAN or NB-IoT benchmark was conducted on the same testbed, the results should be interpreted as a field validation of the proposed architecture rather than as a direct experimental demonstration of superiority over alternative LPWAN systems.

## 1. Introduction

The rapid growth of the global population and the urgent need to improve food production have accelerated the adoption of smart agriculture, where Wireless Sensor Networks (WSNs) and Internet of Things (IoT) technologies are pivotal in enabling precision farming [1,2]. WSNs allow real-time monitoring of soil moisture, crop growth, and environmental conditions, providing data-driven decision-making for irrigation and fertilization [3,4]. Recent advances in sensing, communication, and edge intelligence have further expanded the scope of IoT-enabled smart farming systems [5]. However, ensuring reliable and scalable connectivity across vast, remote agricultural fields remains a critical bottleneck [6].

Conventional LPWAN solutions, such as LoRaWAN, NB-IoT, and Sigfox, have been widely studied and deployed in agricultural IoT scenarios [7,8]. LoRaWAN offers long-range and low-power communication, yet its star-of-stars topology creates a single point of failure at the gateway and limits throughput. NB-IoT provides higher reliability and Quality of Service (QoS), but its dependency on licensed spectrum and subscription fees makes it costly and often impractical for rural regions. Sigfox, while cost-effective, suffers from extremely low data rates, which restrict its applicability for data-intensive applications. Comparative evaluations have shown that LoRaWAN is generally more energy-efficient than NB-IoT, but less capable of supporting large-scale, high-throughput deployments [9,10].

To overcome these challenges, researchers have explored enhancements such as energy-efficient WSN architectures, soft computing for agricultural decision-making, and secure IoT-based frameworks [11]. Recently, multi-hop LoRa networks (LoRaMESH) have been proposed as a means to extend coverage and improve path redundancy by leveraging multi-hop routing and alternate forwarding paths [12,13,14,15]. Studies on underground and linear LoRa-based networks for agriculture and infrastructure monitoring have demonstrated the potential of mesh topologies to improve packet delivery ratio and reduce reliance on a single gateway [12,13]. Likewise, LoRaMESH has shown promising results in critical scenarios, such as earthquake early warning systems, due to its low-latency multi-hop communication [16].

In parallel, Wi-Fi HaLow (IEEE 802.11ah) has emerged as a strong candidate for IoT backhaul, offering native IP connectivity, sub-1 GHz operation for improved range, and support for thousands of devices per access point [15,17,18,19]. Experimental evaluations confirm that Wi-Fi HaLow provides higher throughput and lower latency compared with LoRa, making it suitable for mid-range connectivity in IoT deployments. However, its performance deteriorates over long distances and in obstructed rural environments, which limits its effectiveness as a standalone solution [15].

These findings suggest that neither LoRaMESH nor Wi-Fi HaLow alone can fully address the connectivity requirements of large-scale agricultural deployments. Recent hybrid concepts have combined HaLow and LoRa in other domains, but their objectives and deployment assumptions differ from those of agricultural monitoring systems. In particular, the HaLert arXiv preprint [20] targets post-disaster smart-city resilience, using Wi-Fi HaLow mesh as a high-bandwidth data plane and a LoRa mesh as a control plane, whereas our work focuses on field-scale agricultural sensing, dual-radio cluster gateways, and measured end-to-end operation from sensor nodes to the cloud. Accordingly, this paper contributes through a comprehensive experimental validation of a two-tier LoRaMESH and Wi-Fi HaLow deployment in an outdoor agricultural environment. This study provides measured insights into sensor-to-cloud reliability, distance-dependent backhaul performance, end-node energy consumption, and practical deployment planning limits. The proposed architecture is thus positioned as an engineering integration, with emphasis on real-world performance and applicability for field-scale agricultural sensing.

### Problem Statement and Design Objectives

This paper addresses the following communication problem: how to transport periodic sensing data from battery-powered nodes distributed across large agricultural fields to a cloud backend when direct single-hop gateway connectivity is unreliable and licensed cellular infrastructure may be unavailable or too costly. The target system must therefore balance four competing requirements: wide-area coverage, high delivery reliability, acceptable delay for periodic monitoring, and low operational cost.

The design objectives in this work are:

1. Provide infrastructure-independent sensor-to-cloud connectivity for large rural deployments with mixed line-of-sight and non-line-of-sight propagation conditions.
2. Maintain high end-to-end reliability for periodic environmental sensing while keeping the backhaul architecture simple enough for practical field deployment.
3. Preserve low-duty-cycle operation for end nodes so that long-term battery-powered monitoring remains feasible.
4. Derive deployment guidance from measured results, rather than relying only on simulation or conceptual architecture claims.

The contributions of this paper are as follows:

1. An outdoor agricultural field validation of a hierarchical LoRaMESH and Wi-Fi HaLow deployment over an area of up to 2.5km×1km, focused on practical sensor-to-cloud operation rather than new routing or protocol design.
2. Quantified reliability outcomes for the tested operating regime, with LoRaMESH Packet Delivery Ratios (PDRs) above 90% across one to three hops and Wi-Fi HaLow backhaul PDRs from 99.5% at 50 m to 89.68% at 200 m.
3. Deployment-oriented range guidance derived from the measured data, including a practical LoRaMESH hop spacing of approximately 400–500 m and a degraded but operational Wi-Fi HaLow backhaul regime from 200 m onward under the tested field conditions.
4. A quantified end-node energy profile showing 21.19μA sleep current and battery-life estimates ranging from approximately 19 days at a 2-min sensing interval to approximately 268 days at a 30-min interval.

## 2. Related Work

Wireless connectivity for large-scale agricultural sensing has been widely studied over the past decade. Existing research primarily focuses on low-power wide-area network (LPWAN) technologies, hierarchical wireless sensor network (WSN) architectures, and emerging sub-GHz IP-based backhaul solutions. This section reviews representative studies in these areas and identifies the research gap addressed in this work.

### 2.1. LPWAN Technologies for Agricultural Sensing

Low-power wide-area networks (LPWANs), including LoRaWAN, Sigfox, and NB-IoT, have been extensively adopted in agricultural IoT applications due to their long communication range and low energy consumption [7,21]. LoRaWAN, in particular, enables private network deployment and supports battery-powered sensor nodes operating over several kilometers. However, its star-of-stars topology may introduce coverage limitations in large agricultural environments where direct connectivity between nodes and gateways cannot be guaranteed [8,22].

NB-IoT leverages licensed cellular infrastructure to provide reliable connectivity and enhanced quality of service. Nevertheless, its subscription-based operation and dependence on cellular coverage may increase deployment costs in rural areas [9]. Sigfox offers low-energy operation but provides limited data rates, restricting its applicability in scenarios requiring frequent sensing updates or firmware transmission.

Although LPWAN technologies have demonstrated effectiveness in agricultural monitoring, comparative evaluations indicate that no single LPWAN solution simultaneously optimizes scalability, energy efficiency, throughput, and deployment flexibility for large-scale rural sensing [9,21]. These limitations motivate the exploration of alternative or hybrid network architectures.

### 2.2. Hierarchical and Cluster-Based Sensor Network Architectures

Hierarchical and cluster-based architectures have long been proposed to improve scalability and energy efficiency in WSNs [1]. By organizing nodes into clusters and assigning communication responsibilities to cluster heads or relay nodes, such architectures reduce communication overhead and improve network lifetime [4]. In agricultural IoT systems, hierarchical sensing approaches have been used to optimize data aggregation and improve reliability in distributed deployments [5].

While hierarchical WSN designs provide structural advantages, their performance strongly depends on the underlying communication technology. In large outdoor agricultural environments, the integration of multi-hop sensing and reliable backhaul connectivity remains a key design challenge.

### 2.3. LoRa-Based Multi-Hop Networks

To extend coverage beyond the limitations of star-topology LPWANs, multi-hop LoRa networks, commonly referred to as LoRaMESH, have been investigated in recent studies [12,13,14,15]. By enabling nodes to forward packets through intermediate relays, mesh architectures can provide alternate forwarding paths and extend network coverage in challenging environments.

Experimental deployments in underground sensing systems and linear infrastructure monitoring have demonstrated that mesh-based LoRa communication can enhance packet delivery ratio and delivery performance [12,13]. Moreover, LoRaMESH has been applied in mission-critical scenarios such as earthquake early warning systems, where reliable multi-hop communication is essential [16].

Despite these advances, many reported deployments remain limited in scale or focus on specific infrastructure monitoring applications rather than large-scale agricultural sensing environments.

### 2.4. Wi-Fi HaLow as an IoT Backhaul Technology

Wi-Fi HaLow (IEEE 802.11ah) extends Wi-Fi communication to the sub-1 GHz spectrum, providing improved propagation characteristics compared with traditional 2.4 GHz or 5 GHz Wi-Fi [15,17,18,19]. With native IP support and higher data rates than LoRa-based technologies, Wi-Fi HaLow has been considered for IoT backhaul applications and mid-range connectivity scenarios. Experimental studies have reported that Wi-Fi HaLow achieves higher throughput and lower latency compared with LoRa under similar conditions [15]. However, its performance may degrade in extended non-line-of-sight (NLoS) outdoor environments, and it is rarely used alone as a complete sensing solution in large agricultural deployments.

### 2.5. Hybrid LoRa-Based Architectures

Several hybrid architectures combining LoRa with other wireless technologies have been proposed to overcome the limitations of single-technology IoT systems. For instance, Rivera-Guzman et al. [23] developed a LoRa-based agricultural monitoring system where Wi-Fi was primarily used for local data access at the gateway. Truong [24] investigated a LoRa-Zigbee hybrid approach to enhance local multi-hop communication. Other platforms, such as the ORPHEUS oneM2M living lab reported in an arXiv preprint [25], integrate LoRa with cloud-based infrastructures, often relying on traditional Wi-Fi or wired connectivity for backhaul.

More recently, Ortigoso et al. reported the HaLert architecture in an arXiv preprint [20], which combines Wi-Fi HaLow mesh communication with LoRa mesh networks for smart-city post-disaster scenarios. That work addresses a different domain and objective: Wi-Fi HaLow mesh acts as the primary high-bandwidth data plane, while LoRa mesh supports control-plane and Software-Defined Networking (SDN) resilience functions. By contrast, our study targets agricultural sensing in large outdoor fields, uses dual-radio gateways to bridge LoRaMESH clusters to a Wi-Fi HaLow backhaul, and reports implementation-level measurements from a real agricultural deployment. The novelty of the present work therefore lies in the outdoor agricultural validation and measured engineering evidence rather than in claiming the first-ever hybrid HaLow/LoRa concept.

Table 1 positions the present study against the most relevant peer-reviewed baseline systems in terms of topology, domain, validation type, and reported performance, including a LoRaWAN smart-farming field baseline. The ORPHEUS and HaLert preprints are kept in the narrative because they are conceptually relevant, but they are not used as primary quantitative baselines.

Overall, while previous studies have explored LPWAN technologies, multi-hop LoRa networks, and hybrid architectures independently, there remains a need for real-world experimental validation of hierarchical sensor network designs that integrate multi-hop LoRa sensing with sub-GHz IP-based backhaul in large agricultural environments.

## 3. System Model and Problem Formulation

This section formalizes the deployment setting considered in the paper and clarifies the optimization objective that motivates the proposed architecture. The intent is not to claim a complete cross-layer solution to every deployment variable, but to define the system entities, constraints, and assumptions used throughout the analysis and experimental discussion.

### 3.1. System Model

The target deployment consists of multiple agricultural sensing clusters distributed over a wide rural area. In each cluster, *N* active sensing nodes periodically generate packets of payload size *L* and forward them through a LoRaMESH tier toward a local gateway. Each gateway is equipped with two radios: a LoRa interface for intra-cluster collection and a Wi-Fi HaLow station (STA) for backhaul transmission. All gateways communicate with a central Wi-Fi HaLow access point (AP), which relays aggregated traffic to the cloud backend.

From an end-to-end perspective, one sensing cycle follows the path:

End Node → LoRaMESH Relay/Forwarding Tier → Dual-Radio Gateway → Wi-Fi HaLow AP → Cloud

The design variables that most directly affect performance are the sensing interval $T_{s}$, the LoRa packet airtime $T_{\mathrm{air}}$, the forwarding redundancy $k_{f}$, the number of active nodes *N*, and the physical placement of gateways relative to both sensing nodes and the central AP. These variables jointly influence end-to-end reliability, delay, and energy consumption.

**Table 2:** Main symbols used in the analytical model.

Symbol	Meaning
$T_{s}$	Sensing or packet-generation interval (s)
$\lambda_{s}$	Packet generation rate per node, with $\lambda_{s}=1/T_{s}$
*N*	Number of active sensing nodes in one LoRaMESH cluster
*L*	Payload size (bits per packet)
$k_{f}$	Average forwarding factor, i.e., average number of transmissions per original packet
$T_{\mathrm{air}}$	Airtime of one LoRa packet under the configured PHY parameters
*G*	Normalized channel load, approximated by $G=N\lambda_{s}T_{\mathrm{air}}k_{f}$
$P_{t}$	LoRa transmit power (dBm)
$G_{t},G_{r}$	Transmit and receive antenna gains (dBi)
$P_{r}\left(d\right)$	Received power for an inter-hop distance *d* (dBm)
PL(d)	Path loss at distance *d* (dB)
*n*	Log-distance path-loss exponent
$\sigma_{\mathrm{sh}}$	Shadow-fading standard deviation (dB)
$R^{2}$	Coefficient of determination for the least-squares propagation fit
$M_{f}$	Link-margin term accounting for fading and implementation losses (dB)
γ	Received Signal-to-Interference-plus-Noise Ratio (SINR)
$I_{\mathrm{co}}$	Co-channel interference power
$N_{0}BF$	Effective receiver noise power term
$C_{\mathrm{Sh}}$	Shannon capacity upper bound $B\log_{2}\left(1+\gamma \right)$ (bit/s)
$S_{\mathrm{app}}$	Effective application-layer throughput (bit/s)
$S_{\mathrm{occ}}$	Occupancy-limited throughput bound (bit/s)
$P_{\mathrm{busy}}$	Probability that the channel is sensed busy before transmission
$P_{\mathrm{res}}$	Residual collision probability due to hidden terminals and overlapping backoff expiration
$P_{\mathrm{succ}}$	Approximate successful-transmission probability under LBT

### 3.2. Link Budget, Path Loss, and SINR Model

For the LoRa tier, the received power between two consecutive hops separated by distance *d* is denoted by $P_{r}\left(d\right)$ and is expressed in dB form as:

(1) $$P_{r}\left(d\right)=P_{t}+G_{t}+G_{r}-PL\left(d\right)-M_{f},$$

where $P_{t}$ is the configured transmit power, $G_{t}$ and $G_{r}$ are transmitter and receiver antenna gains, and $M_{f}$ is a lumped margin accounting for fading and implementation losses. To interpret the outdoor measurements, the large-scale path loss can be modeled by the standard log-distance relation

(2) $$PL\left(d\right)=PL\left(d_{0}\right)+10n\log_{10}\;\left(\frac{d}{d_{0}}\right)+X_{\sigma },$$

where $d_{0}$ is a reference distance, *n* is the path-loss exponent, and $X_{\sigma }∼N\left(0,\sigma_{\mathrm{sh}}^{2}\right)$ captures log-normal shadowing. In the present measurement campaign, line-of-sight (LoS) and non-line-of-sight (NLoS) points were mixed within the same outdoor route; therefore, any fitted *n* or $\sigma_{\mathrm{sh}}$ should be interpreted as an aggregate field descriptor rather than as a class-specific propagation model.

For a candidate reception event, the corresponding Signal-to-Interference-plus-Noise Ratio (SINR) is

(3) $$\gamma =\frac{P_{r}}{I_{\mathrm{co}}+N_{0}BF},$$

where $I_{\mathrm{co}}$ denotes co-channel interference power, $N_{0}$ is the thermal-noise density, *B* is the receiver bandwidth, and *F* is the receiver noise factor. Successful reception requires both an adequate received power margin and a sufficiently large SINR with respect to the selected LoRa PHY configuration.

For completeness, the Shannon capacity upper bound of an equivalent narrowband link is

(4) $$C_{\mathrm{Sh}}=B\log_{2}\left(1+\gamma \right),$$

which serves here as an upper envelope rather than as the actual LoRa throughput, since the measured system is constrained by fixed packet airtime, LBT access, and multi-hop relaying overhead.

### 3.3. Problem Formulation

The communication problem can be written as the search for a deployment and operating regime that maximizes cloud-level delivery reliability while respecting delay, energy, and scalability constraints:

(5) $$\max\;{\mathrm{PDR}}_{\mathrm{cloud}}$$

subject to

(6) $$\bar{\Delta t}\leq \Delta_{\max},$$

(7) $$I_{\mathrm{avg}}\leq I_{\max},$$

(8) $$G=N\lambda_{s}T_{\mathrm{air}}k_{f}<G_{\max}.$$

Here, $\bar{\Delta t}$ is the mean end-to-end latency, $I_{\mathrm{avg}}$ is the average end-node current consumption, and *G* is the normalized LoRa channel load. The bound $\Delta_{\max}$ denotes the maximum tolerable end-to-end delay budget for the sensing application, $I_{\max}$ denotes the maximum allowable average current consistent with the target battery lifetime, and $G_{\max}$ denotes the admissible normalized-load ceiling used to keep the LoRaMESH tier in a non-congested operating regime. In practical terms, the objective is to keep sensing traffic reliable and energy-efficient without pushing the LoRaMESH tier into a congestion-dominated regime or requiring dense wired or cellular infrastructure.

### 3.4. Assumptions

The analytical and experimental interpretation in this paper is based on the following assumptions:

- Sensor traffic is periodic and generated at a common interval $T_{s}$ during each experiment.
- All LoRa packets use the same configured PHY parameters within a given experiment, so $T_{\mathrm{air}}$ is treated as fixed for the analytical load model.
- The forwarding factor $k_{f}$ captures the average redundancy introduced by controlled flooding and is used as an aggregate parameter rather than a per-link routing variable.
- The approximation $P_{\mathrm{busy}}\approx G$ is intended only for the moderate-load regime considered in the paper and is not claimed as a universal collision model.
- The Wi-Fi HaLow backhaul is modeled as a star topology in which each gateway communicates with a single central AP.
- Once a packet reaches the AP, the remaining AP-to-cloud path is assumed to be available and is not modeled as the dominant source of loss in the field experiments.
- The battery lifetime analysis applies to end nodes only. Relay nodes and gateway nodes are supplied by continuous external power in the current design and are therefore excluded from battery lifetime estimation.

## 4. System Architecture

To address the connectivity limitations of large-scale agricultural deployments, a two-tier hierarchical wireless sensor network architecture is proposed. The system integrates a multi-hop LoRa-based sensing tier with a Wi-Fi HaLow backhaul tier to enable scalable communication across distributed farming areas.

### 4.1. Overall Architecture

**Figure 1:**
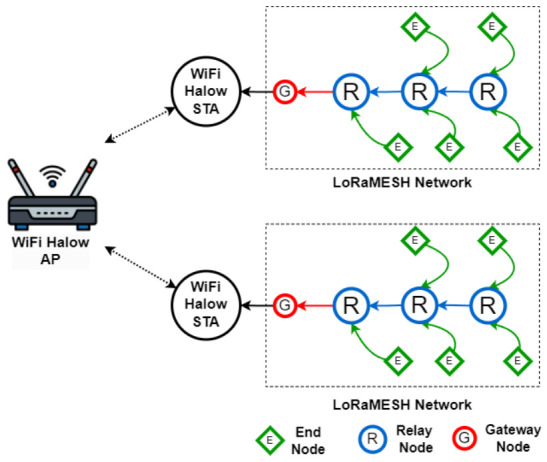
Hybrid Wi-Fi HaLow and LoRaMESH system architecture.

The overall architecture is illustrated in Figure 1. The system is organized into two functional tiers. The first tier consists of LoRaMESH sensor clusters deployed across agricultural fields. Within each cluster, sensor nodes form a multi-hop mesh network using the LoRa physical layer. This configuration enables distributed sensing nodes to forward data through intermediate relays when direct communication with the cluster gateway is not feasible.

The second tier provides backhaul connectivity using Wi-Fi HaLow (IEEE 802.11ah). Each cluster is connected to a dual-radio gateway integrating a LoRa transceiver and a Wi-Fi HaLow station (STA). The gateways communicate with a central HaLow access point (AP), which aggregates data and forwards it to a cloud backend via available Internet uplinks. This hierarchical organization separates local sensing communication from long-range IP-based aggregation, improving scalability and deployment flexibility in large rural environments.

### 4.2. LoRaMESH Sensing Tier

The sensing tier employs a multi-hop LoRa-based mesh topology to provide last-mile connectivity among distributed sensor nodes. Compared with conventional LoRaWAN star topologies, the mesh arrangement allows intermediate nodes to relay packets, improving coverage and path redundancy in environments with irregular terrain or vegetation obstruction. Each cluster operates autonomously, with one node designated as the gateway for backhaul communication. The mesh design prioritizes reachability and potential path redundancy over strict bandwidth optimization, reflecting the low data-rate characteristics of agricultural sensing workloads.

### 4.3. Wi-Fi HaLow Backhaul Tier

The backhaul tier utilizes Wi-Fi HaLow (IEEE 802.11ah) to aggregate traffic from multiple LoRaMESH clusters. Operating in the sub-1 GHz band, Wi-Fi HaLow provides improved propagation characteristics compared with conventional Wi-Fi while maintaining native IP connectivity. Each cluster gateway connects to a central HaLow access point, forming a star-based backhaul network. The access point forwards aggregated data to cloud services for storage and analysis. This separation of sensing and backhaul layers reduces infrastructure dependency while enabling integration with IP-based services.

## 5. Materials and Methods

**Figure 2:**
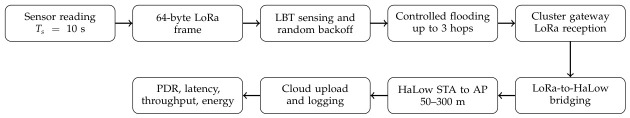
End-to-end methodology and data-flow sequence used in the field evaluation. The LoRaMESH tier used a fixed 64-byte frame, a 10 s packet interval, LBT channel access with randomized backoff, and controlled flooding bounded by a three-hop TTL. The gateway then forwarded received packets over the Wi-Fi HaLow backhaul to the cloud, where PDR, latency, throughput, channel, and energy metrics were extracted.

Figure 2 summarizes the end-to-end experimental workflow used in this study, from packet generation at the sensor node through LoRaMESH forwarding, gateway bridging, Wi-Fi HaLow backhaul transmission, cloud logging, and metric extraction.

### 5.1. Hardware Platform

**Figure 3:**
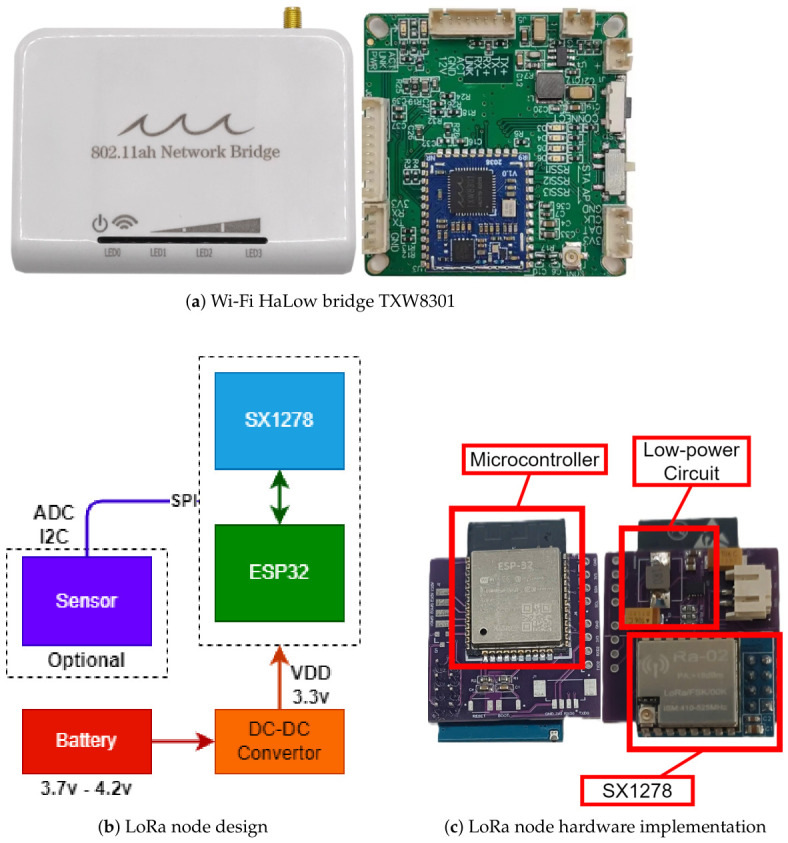
(**a**) Wi-Fi HaLow bridge TXW8301, (**b**) LoRa node design, and (**c**) LoRa node hardware implementation.

All LoRaMESH sensor and relay nodes are implemented using an ESP32 microcontroller combined with an SX1278 LoRa transceiver (Figure 3). The ESP32 provides dual-core processing capability and low-power operation modes, while the SX1278 supports programmable spreading factors and sub-1 GHz communication. Cluster gateways integrate both the SX1278 LoRa module and a TXW8301 Wi-Fi HaLow module, enabling dual-radio operation. In the current deployment design, only end nodes are battery-powered; relay nodes and dual-radio gateways are supplied by continuous external power because they perform forwarding, listening, and backhaul duties that differ from the low-duty-cycle sensing role.

### 5.2. Wi-Fi HaLow Backhaul Configuration and Reproducibility Boundary

The backhaul tier used the TXW8301 Wi-Fi HaLow platform in a one-AP/multiple-STA star topology, with each gateway operating as a station and the central collection point operating as the access point.

For reproducibility, the available HaLow information is separated into three categories. First, the directly measured and confirmed experimental parameters are the TXW8301 platform family, the one-AP/multiple-STA topology, gateway-to-AP distances of 50, 100, 200, and 300 m, 1000 forwarded packets per cluster, and the available TXW configuration record: operation over 866–867 MHz with 1 MHz channel width, Wi-Fi MCS0, 17 dBm transmit power, and TCP port 8001 for the forwarding test. A recovered device-status snapshot also reports channel 3 at 908.0 MHz with 8 MHz local bandwidth, a background scan list of 908.0, 916.0, and 924.0 MHz, transmit-link status of MCS1 with 2 MHz bandwidth, receive-link status of MCS7 with 8 MHz bandwidth, measured SNR values of approximately 42–44 dB, and measured RSSI of approximately −32 to −36 dBm. Because this status snapshot is not a complete per-trial configuration log, it is used only to document the observed module state and not to claim a fixed PHY setting for every HaLow trial. Second, the parameters known only from the TX-AH-Rx00P/TXW830x module documentation are the supported sub-GHz operating range, 1/2/4/8 MHz channel bandwidth options, and nominal PHY rates from 150 kb/s to 32.5 Mb/s. These values describe the module-family capability rather than additional confirmed per-trial settings. Third, the archived field logs do not preserve the antenna gain, firmware build, exact rate-adaptation behavior across all packets, or whether Restricted Access Window (RAW) or Target Wake Time (TWT) was enabled. The HaLow results are therefore reproducible at the platform, topology, distance, packet-count, measured-PDR, available TXW configuration, and device-status-snapshot level, but not at the complete PHY/MAC implementation level.

### 5.3. LoRa Communication Parameters

The LoRa communication layer was configured using the following parameters: transmission power of 20 dBm, spreading factor SF7, bandwidth of 125 kHz, coding rate 4/5, and a fixed packet interval of 10 s per transmission. The selected configuration represents a trade-off between airtime efficiency and communication reliability for mid-range agricultural deployments. SF7 was chosen to reduce airtime and energy consumption while maintaining acceptable link reliability within the tested range. A bandwidth of 125 kHz was adopted to balance sensitivity and throughput. The coding rate 4/5 provides forward error correction capability without excessive redundancy. The maximum hop count in the LoRaMESH network was limited to three hops.

### 5.4. Packet Structure

A fixed 64-byte frame format is adopted to ensure predictable airtime and processing overhead in multi-hop LoRa communication. The frame includes source and destination identifiers, a message sequence number for duplicate suppression, a time-to-live (TTL) field to bound propagation depth, a data payload field, and a checksum for integrity verification. When encryption is enabled, the payload is protected using AES-128 in Counter mode (AES-128-CTR) at the application layer with a pre-shared fixed 128-bit symmetric key. To ensure keystream uniqueness, a per-packet nonce/counter is constructed by combining the device identifier and a monotonically increasing message sequence number, preventing reuse across transmissions. The archived experiment record does not provide a complete security configuration record for session key generation, key rotation, or authenticated integrity protection Consequently, the reported 2-byte checksum should be interpreted only as a corruption-detection field and not as a cryptographic integrity or authenticity guarantee. The fixed-size format ensures deterministic behavior and simplifies duplicate detection within flooding-based routing.

### 5.5. Security Scope

The present study evaluates communication performance rather than providing a complete security validation. Two security layers are relevant. First, the LoRaMESH tier supports AES-CTR payload encryption at the application layer, which provides a confidentiality mechanism for the test payloads but does not by itself provide message authentication. Second, the Wi-Fi HaLow backhaul inherits the security mechanisms available in the underlying IEEE 802.11ah stack. However, the archived field-trial logs do not preserve a fully auditable security configuration, the key-distribution procedure, the authentication configuration, replay-protection settings, or Wi-Fi HaLow security profile used in each trial. The current paper therefore does not experimentally evaluate key management, mutual authentication, authenticated integrity protection, replay resistance, Sybil resistance, or denial-of-service resistance, and it does not claim that the checksum alone provides cryptographic integrity. These aspects are treated as out of scope for the present field trial and are explicitly identified as future hardening work.

### 5.6. Routing Mechanism

Intra-cluster communication is based on a lightweight controlled flooding protocol. Upon generating or receiving a packet, a node verifies whether the packet has been previously processed using the combination of source identifier and sequence number. If the packet is new, it is rebroadcast once after a randomized backoff interval to reduce collision probability. Otherwise, it is discarded. A TTL field limits the maximum hop count, preventing uncontrolled propagation. This approach avoids routing table maintenance while maintaining reachability in sparse agricultural deployments.

### 5.7. Deployment Scenarios

The proposed architecture is applicable to large open farms, orchards, greenhouses, and distributed agricultural cooperatives. The mesh topology supports coverage in obstructed environments, while the Wi-Fi HaLow backhaul enables regional data aggregation without cellular dependency.

## 6. Analytical Load and Collision Model

To complement the field experiments and provide a generalized interpretation of the observed performance, this section introduces an analytical framework describing traffic load, scalability behavior, and collision tendency in the proposed LoRaMESH tier under Listen-Before-Talk (LBT) channel access. The objective of this analysis is not to derive an exact closed-form model for all deployment conditions, but to provide a tractable approximation that explains the measured Packet Delivery Ratio (PDR) and latency trends and clarifies scalability limits for large-scale agricultural deployments. All analytical expressions in this section are therefore used as approximate design-guidance tools rather than fully validated predictive models. This framing is important because parameters such as the forwarding factor $k_{f}$, hidden-terminal geometry, and the approximation $P_{\mathrm{busy}}\approx G$ were not independently measured for every packet in the field trial.

### 6.1. LoRa Airtime Under the Tested Configuration

Under the configured LoRa PHY setting (SF7, 125 kHz, CR 4/5, explicit header, CRC enabled, 64-byte packet), the symbol duration is

(9) $$T_{\mathrm{sym}}=\frac{2^{SF}}{BW}=\frac{2^{7}}{125000}\approx 1.024\;\mathrm{ms}.$$

Using the standard LoRa payload-symbol expression for the stated configuration gives approximately 103 payload symbols. With an 8-symbol preamble, the resulting airtime is

(10) $$T_{\mathrm{air}}\approx \left(8+4.25+103\right)T_{\mathrm{sym}}\approx 118\;\mathrm{ms}.$$

This measured-configuration airtime is used in the subsequent load and throughput projections.

### 6.2. Traffic Model

In typical smart agriculture scenarios, sensor nodes generate periodic sensing traffic. Let $T_{s}$ denote the packet interval (seconds per packet). The packet generation rate per node is therefore:

(11) $$\lambda_{s}=\frac{1}{T_{s}}.$$

For a LoRaMESH cluster consisting of *N* active sensing nodes, the aggregate offered traffic rate is:

(12) $$\Lambda_{\mathrm{cluster}}=N\lambda_{s}.$$

Let *L* denote the payload size (bits per packet). The raw data rate injected into the cluster becomes:

(13) $$R_{\mathrm{cluster}}=N\lambda_{s}L.$$

Because the proposed LoRaMESH uses controlled flooding with relay redundancy, each packet may be retransmitted multiple times by intermediate nodes. Let $k_{f}$ denote the average forwarding factor (i.e., the average number of transmissions per original packet). The effective channel load therefore becomes:

(14) $$R_{\mathrm{eff}}=k_{f}R_{\mathrm{cluster}}=k_{f}N\lambda_{s}L.$$

This expression highlights that the effective load grows linearly with both node density and forwarding redundancy.

### 6.3. Scalability Analysis

To evaluate scalability, it is useful to express channel occupancy in normalized form. Let $T_{\mathrm{air}}$ denote the airtime of a single LoRa packet under the configured parameters (SF7, 125 kHz, CR 4/5). The normalized channel load *G* can be approximated using Equation (8). Here, *G* represents the fraction of time the channel is occupied by transmissions. When G≪1, the network operates in a sparse-load regime, and contention is minimal. As *G* approaches unity, the probability of channel deferral and collision increases significantly.

Substituting the tested configuration into Equation (8) yields

(15) $$G=N\left(\frac{1}{10}\right)\left(0.118\right)k_{f}=0.0118\;N\;k_{f}.$$

For the sparse controlled-flooding regime observed in the field, we use a representative planning value of $k_{f}\approx 1.3$. This value was not directly measured from per-packet forwarding logs; rather, it represents a modest relay-reuse assumption in which most packets are delivered with one transmission round and a minority require one additional relay-forwarding event. It is therefore used only for sensitivity-based planning, not as a validated invariant of the protocol. With this assumption:

(16) G≈0.0118N×1.3≈0.015N.

This formulation reveals two important scalability properties:

- The load increases linearly with the number of sensing nodes *N*.
- Flooding redundancy ($k_{f}>1$) amplifies channel occupancy beyond the nominal sensing rate.

Therefore, while controlled flooding improves reachability in sparse rural deployments, it may limit scalability in dense scenarios due to increased effective airtime consumption. The current field deployment corresponds to a low-to-moderate load regime, as reflected by the measured PDR above 90%. However, the analytical model indicates that denser clusters or shorter packet intervals would increase *G*, potentially pushing the network toward congestion-dominated behavior.

Table 3 shows that the projected non-congested cluster size is sensitive to forwarding redundancy. The practical implication is that the previously cited 30–35 node range should be read as a planning result for a sparse deployment with modest relay reuse, not as a universal hard limit.

### 6.4. Throughput Bound

The effective application-layer throughput of the LoRaMESH tier can be written as

(17) $$S_{\mathrm{app}}=N\lambda_{s}L\cdot \mathrm{PDR},$$

while an occupancy-limited cluster throughput bound is

(18) $$S_{\mathrm{occ}}\leq \frac{G_{\max}L}{T_{\mathrm{air}}k_{f}}.$$

Using L=512 bits, $T_{\mathrm{air}}\approx 118$ ms, $k_{f}\approx 1.3$, and a conservative non-congested operating target of $G_{\max}=0.5$, the resulting cluster-level bound is approximately

(19) $$S_{\mathrm{occ}}≲1.67\;\mathrm{kb}/s.$$

This bound clarifies that, under the traffic rates considered here, the LoRaMESH sensing tier is the first scalability bottleneck, whereas the Wi-Fi HaLow backhaul remains comfortably above the required aggregate sensor throughput.

### 6.5. Collision Model Under Listen-Before-Talk

The proposed LoRaMESH employs a Listen-Before-Talk (LBT) mechanism combined with randomized backoff. Before transmission, a node senses the channel and defers its transmission if ongoing activity is detected. This reduces direct collisions compared with pure ALOHA-based random access. Under LBT operation, the probability of successful transmission can be approximated as:

(20) $$P_{\mathrm{succ}}=\left(1-P_{\mathrm{busy}}\right)\left(1-P_{\mathrm{res}}\right),$$

where:

- $P_{\mathrm{busy}}$ is the probability that the channel is sensed busy prior to transmission,
- $P_{\mathrm{res}}$ is the residual collision probability caused by hidden terminals and overlapping backoff expiration.

For moderate traffic loads, $P_{\mathrm{busy}}$ can be approximated as proportional to the normalized load:

(21) $$P_{\mathrm{busy}}\approx G.$$

Residual collisions arise from two main sources:

- **Hidden-node collisions**, where two nodes cannot sense each other but interfere at a common receiver.
- **Simultaneous channel access**, when multiple nodes sense the channel idle and initiate transmission within overlapping backoff intervals.

Although LBT significantly reduces direct contention, it does not eliminate these residual effects. As node density increases, both $P_{\mathrm{busy}}$ and $P_{\mathrm{res}}$ are expected to grow, reducing $P_{\mathrm{succ}}$ and increasing latency due to channel deferrals. This analytical interpretation explains the experimentally observed behavior: the high PDR values measured in the field indicate that the network operates below the congestion threshold, while the moderate latency reflects both LoRa airtime and LBT-induced deferral.

Overall, the analysis indicates that the proposed architecture is well suited for sparse and moderately dense agricultural deployments, but would require more selective routing or adaptive forwarding strategies for very high node densities.

## 7. Experimental Setup

To validate the performance of the proposed hierarchical LoRaMESH–Wi-Fi HaLow architecture, a series of controlled outdoor experiments were conducted under real agricultural conditions. The evaluation focused on (i) standalone multi-hop LoRaMESH performance and (ii) end-to-end performance of the integrated hybrid system from sensor nodes to the cloud. All measurements were performed in mixed line-of-sight (LoS) and non-line-of-sight (NLoS) environments characterized by open fields, vegetation, and moderate terrain irregularities.

### 7.1. Deployment Environment

**Figure 4:**
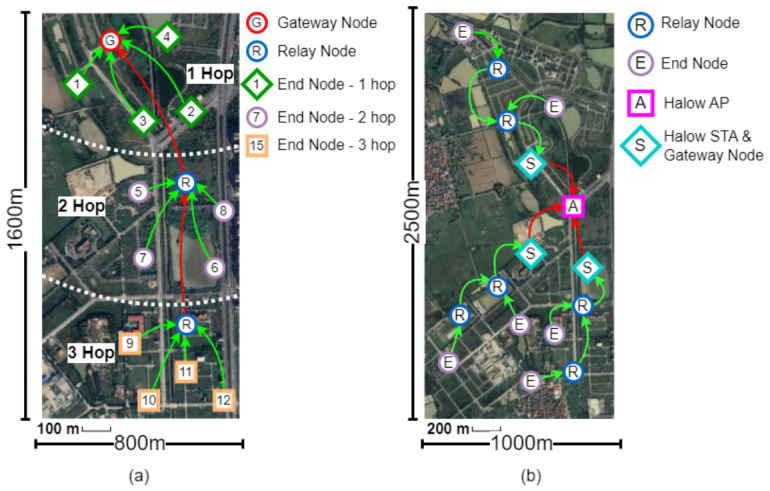
(**a**) LoRaMESH network experiment and (**b**) hybrid network experiment. Base aerial imagery source: Google Earth/Google Maps imagery; the base layer is used only for site-layout context and should be replaced with an original schematic if reuse permission cannot be confirmed before publication.

The physical layouts of both experimental deployments are illustrated in Figure 4. Figure 4a shows the standalone LoRaMESH experiment, in which a single Gateway Node (G), two Relay Nodes (R), and twelve End Nodes are arranged in a deterministic linear topology spanning a 1600m×800m area. The nodes are organized into three hop tiers: End Nodes 1–4 communicate directly with the Gateway (1-hop), End Nodes 5–8 reach it through one Relay (2-hop), and End Nodes 9–12 require two intermediate Relays (3-hop), with approximately 500m separation between successive relay stages. Figure 4b shows the large-scale hybrid deployment, in which three independent LoRaMESH clusters are distributed across a 2500m×1000m agricultural field. Each cluster contains its own Gateway Node equipped with a Wi-Fi HaLow station (S), multiple Relay Nodes, and several End Nodes, with approximately 500m spacing between End Nodes and their nearest Relay Nodes. The three HaLow stations connect to a single centralized HaLow Access Point (A) in a star backhaul topology, with gateway-to-AP distances ranging from 50m to 300m. Environmental conditions during testing included moderate wind and ambient temperatures between 22 and 28 °C.

### 7.2. Standalone LoRaMESH Deployment

To evaluate intrinsic multi-hop reliability, a deterministic 3-hop linear topology was deployed. Each End Node transmitted 200 packets of 64 bytes.

Packet Delivery Ratio is defined as:

(22) $$\mathrm{PDR}=\frac{N_{\mathrm{rx}}}{N_{\mathrm{tx}}}\times 100\%$$

where $N_{\mathrm{tx}}$ is the number of transmitted packets and $N_{\mathrm{rx}}$ is the number successfully received at the Gateway.

For each packet *i*, latency is defined as:

(23) $$\Delta t_{i}=t_{i}^{\mathrm{rx}}-t_{i}^{\mathrm{tx}}$$

The average latency is computed as:

(24) $$\bar{\Delta t}=\frac{1}{N_{\mathrm{rx}}}\sum_{i=1}^{N_{\mathrm{rx}}}\Delta t_{i}$$

The standard deviation of latency is given by:

(25) $$\sigma =\sqrt{\frac{1}{N_{\mathrm{rx}}-1}\sum_{i=1}^{N_{\mathrm{rx}}}{\left(\Delta t_{i}-\bar{\Delta t}\right)}^{2}}$$

Received Signal Strength Indicator (RSSI) and Signal-to-Noise Ratio (SNR) values were recorded for each received packet. Measurements were collected at distances ranging from 50 m to 600 m. These metrics enable evaluation of path loss behavior and demodulation reliability under real propagation conditions.

### 7.3. Large-Scale Hybrid Deployment

To evaluate the full hierarchical architecture, three independent LoRaMESH clusters were deployed as depicted in Figure 4b. Each Gateway Node was equipped with a Wi-Fi HaLow station (STA) using a TXW8301 module. All gateways communicated with a central HaLow Access Point (AP) arranged in a star topology. The end-to-end communication path was:

End Node → LoRaMESH → Gateway → HaLow STA → HaLow AP → Cloud

To evaluate hybrid-network delivery reliability, each cluster transmitted 1000 consecutive packets to the cloud. Measurements were performed at gateway-to-AP distances of 50 m, 100 m, 200 m, and 300 m.

Cloud-level PDR is defined as:

(26) $${\mathrm{PDR}}_{\mathrm{cloud}}=\frac{N_{\mathrm{cloud}}}{N_{\mathrm{tx}}}\times 100\%$$

where $N_{\mathrm{cloud}}$ is the number of packets successfully received by the cloud server.

### 7.4. Energy Measurement Procedure

Energy consumption of LoRa end nodes was measured using the Nordic Power Profiler Kit II (PPK2) (Nordic Semiconductor, Trondheim, Norway), which provides high-resolution current sampling suitable for low-power IoT devices. The measurement setup supplied a regulated DC voltage of 4.0 V to the device under test, corresponding to the nominal voltage of a single-cell LiPo battery typically used in agricultural sensor nodes. The PPK2 was configured in source-measure mode, allowing simultaneous power-supply and current monitoring. Current samples were recorded with microampere-level resolution during different operational phases, including deep sleep, wake-up, radio transmission, and reception. Sleep current was measured over a 60 s interval to capture steady-state consumption and filter transient spikes associated with internal clock calibration. Transmission current was averaged over multiple packet transmissions to account for variations caused by random backoff and channel access delay. The transmission profile included microcontroller unit wake-up, LoRa radio initialization, payload transmission, and return to sleep state.

Energy per transmission event was computed as:

(27) $$E_{\mathrm{tx}}=V\int_{0}^{T_{\mathrm{tx}}}I\left(t\right)\;dt,$$

where V=4 V is the supply voltage, I(t) is the instantaneous current, and $T_{\mathrm{tx}}$ is the transmission duration. Battery lifetime estimation was derived only for battery-powered end nodes based on the measured duty cycle and a nominal 2400 mAh LiPo battery capacity. Using the same packet interval notation $T_{s}$ defined in Table 2, the average current consumption was computed as:

(28) $$I_{\mathrm{avg}}=\frac{E_{\mathrm{cycle}}}{VT_{s}},$$

where $E_{\mathrm{cycle}}$ represents the total energy consumed in one sensing-transmission cycle. This procedure estimates long-term autonomous operation for end nodes under periodic agricultural sensing workloads. Relay nodes and gateway nodes are excluded from this battery lifetime calculation because they are externally powered in the current design.

### 7.5. Reproducibility Considerations

All experiments were conducted under consistent radio configuration and environmental conditions. Packet transmission intervals, hop count limits, and backoff settings were kept constant across trials. Measurement scripts and logging mechanisms were standardized to ensure repeatability.

## 8. Results

This section presents the experimental performance of the proposed hierarchical architecture based on two groups of field experiments: (i) standalone LoRaMESH evaluation over a 1600m×800m deployment and (ii) hybrid Wi-Fi HaLow + LoRaMESH evaluation over a 2500m×1000m area. The reported results include packet delivery ratio (PDR), end-to-end latency, effective throughput, backhaul reliability, energy consumption, and channel characterization metrics.

### 8.1. LoRaMESH Reliability

**Figure 5:**
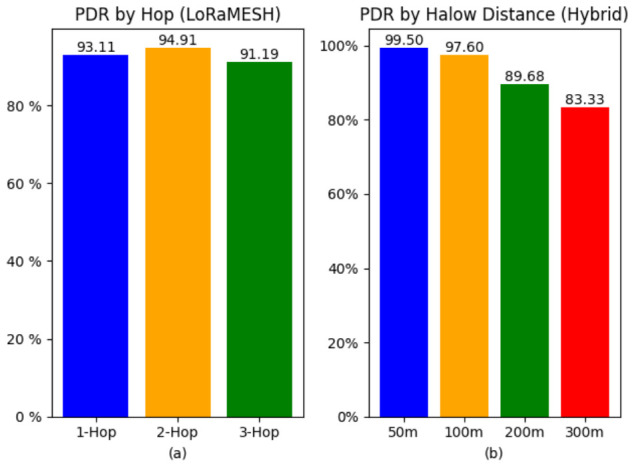
(**a**) PDR by hop count in the LoRaMESH network, (**b**) PDR by Wi-Fi HaLow backhaul distance in the hybrid network.

The standalone LoRaMESH deployment was configured as a linear three-hop topology with approximately 500 m separation between nodes. Each End Node transmitted 200 encrypted 64-byte packets toward the Gateway Node. The measured PDR values were 93.11%, 94.91%, and 91.19% for one-hop, two-hop, and three-hop configurations, respectively (Figure 5a). Across all tested hop counts, PDR remained above 90%, indicating stable multi-hop communication under mixed LoS/NLoS agricultural conditions. In terms of the system model, these PDR values are the experimental response variable associated with Equation (5), and they indicate that the tested hop spacing retained adequate received power and SINR margins as represented by Equations (1) and (3). The slightly higher PDR observed for the two-hop case compared with the one-hop case is interpreted as measurement variability rather than a systematic gain from additional relaying, because each configuration used a limited packet sample and experienced different instantaneous interference and propagation conditions in the field.

### 8.2. LoRaMESH Latency

Latency was computed from timestamp differences between packet transmission and successful reception at the Gateway. Table 4 summarizes the statistical delay characteristics for different hop counts. The measured mean latency values were 10.62 s, 10.81 s, and 13.31 s for one-hop, two-hop, and three-hop configurations, respectively. The corresponding standard deviations were 5.13 s, 4.93 s, and 6.72 s. These standard deviations are used as a jitter indicator for the present periodic-traffic experiment, while packet-to-packet inter-arrival jitter was not logged separately. The observed minimum–maximum delay ranges were 4.62–33.56 s (one-hop), 2.31–23.14 s (two-hop), and 3.10–43.64 s (three-hop). These measurements provide the empirical latency term used to interpret the delay constraint in Equation (6) for periodic environmental monitoring. Although latency variability increased with hop count, the average delay remained within a range suitable for periodic environmental monitoring applications.

### 8.3. Effective Throughput

With a fixed packet size of 64 bytes (L=512 bits) and a transmission interval of $T_{s}=10$ s, the per-node offered application rate is 51.2 bit/s. This substitution instantiates the packet-rate and raw traffic terms in Equations (11) and (13). Combining this rate with the measured PDR values using Equation (17) yields effective delivered throughputs of approximately 47.67 bit/s, 48.59 bit/s, and 46.69 bit/s for the one-hop, two-hop, and three-hop cases, respectively. The narrow spread confirms that the tested LoRaMESH operating point remained in a lightly loaded regime despite the additional forwarding overhead.

For context, the nominal LoRa PHY bit rate for SF7, 125 kHz bandwidth, and coding rate 4/5 is approximately $R_{\mathrm{PHY}}=SF\times BW/2^{SF}\times 4/5\approx 5.47$ kb/s. The achieved per-node delivered application rate of 46.69–48.59 bit/s is therefore only about 0.85–0.89% of this nominal PHY rate. This gap is expected because the experiment used a 10 s sensing interval, fixed 64-byte payloads, packet headers, LBT/backoff, and multi-hop forwarding overhead; the metric reported here is delivered application throughput, not continuous PHY-layer capacity.

At the projected cluster scale, the application throughput remains modest. For approximately 30–32 active nodes, the aggregate delivered sensing rate is on the order of 1.4–1.6 kb/s, which is well below the throughput capability commonly reported for Wi-Fi HaLow backhaul links in the literature. This supports the analytical conclusion that LoRa airtime occupancy, rather than HaLow capacity, is the dominant throughput constraint in the present architecture. Because the experiments used the same packet size and reporting interval for all sources, no strong fairness imbalance is visible at the aggregate level. Nevertheless, node-level fairness was not explicitly measured, and hidden-terminal geometry may still disadvantage some relay paths in denser deployments.

### 8.4. Hybrid Wi-Fi HaLow Backhaul Performance

The hybrid deployment included three LoRaMESH clusters connected via Wi-Fi HaLow backhaul to a centralized Access Point. To evaluate backhaul-layer reliability, packets successfully reaching each Gateway Node were forwarded to the cloud, and PDR was measured at varying gateway-to-AP distances. At 50 m and 100 m, the measured HaLow PDR values were 99.5% and 97.6%, respectively (Figure 5b). At extended distances, PDR values of 89.68% (200 m) and 83.33% (300 m) were observed. These distance-dependent backhaul measurements constrain the practical gateway-placement interpretation of the same link-budget and path-loss planning logic introduced in Equations (1) and (2), although detailed HaLow PHY parameters were not preserved in the archived logs. For each cluster, 1000 packets were transmitted toward the cloud. The resulting end-to-end cloud-level PDR closely followed the HaLow-layer reliability trends at corresponding distances.

### 8.5. Energy Consumption of LoRa End Nodes

**Figure 6:**
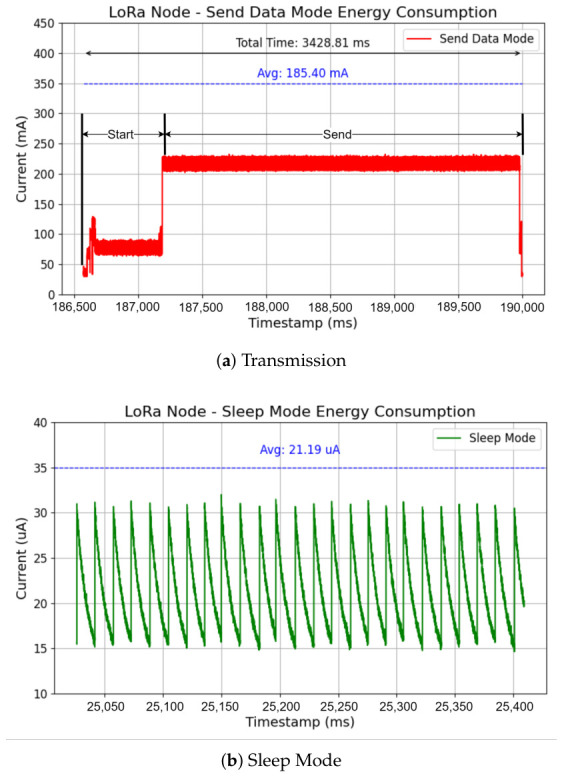
Energy consumption of the end node in (**a**) transmission and (**b**) sleep mode.

Energy consumption was measured using the Nordic Power Profiler Kit II (PPK2). In sleep mode, the end node exhibited an average current consumption of 21.19μ A, measured over a 60 s interval. During LoRa transmission, the end node consumed an average current of 185.40mA for approximately 3.43s, including wake-up and radio initialization phases (Figure 6). Given a transmission interval of 10 s per packet during testing, transmission represents a small fraction of the end-node duty cycle. Under typical agricultural sensing intervals (several minutes), sleep-mode operation dominates end-node energy consumption. These measurements provide the end-node current term used to interpret the energy constraint in Equation (7).

Table 5 maps the measured end-node duty cycle to practical reporting intervals. These lifetime estimates apply only to battery-powered end nodes and should not be generalized to relay or gateway roles.

### 8.6. Channel Characterization: RSSI and SNR

**Figure 7:**
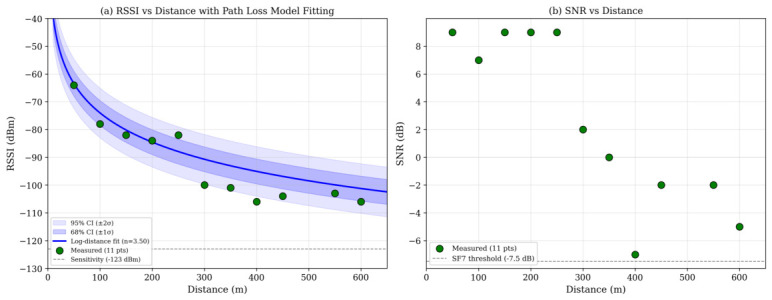
Measured RSSI and SNR values as a function of transmission distance under mixed LoS/NLoS agricultural conditions. (**a**) RSSI versus distance with a least-squares log-distance path-loss fit (n=3.50), approximate 68% ($\pm 1\sigma_{\mathrm{sh}}$) and 95% ($\pm 2\sigma_{\mathrm{sh}}$) shadowing bands derived from $\sigma_{\mathrm{sh}}=4.47$ dB, and the SF7 receiver sensitivity threshold (−123 dBm). (**b**) SNR versus distance and the SF7 demodulation threshold (−7.5 dB).

RSSI and SNR values were recorded at distances ranging from 50 m to 600 m under mixed LoS/NLoS agricultural conditions (Figure 7). RSSI decreased progressively with increasing distance, from approximately −64 dBm at 50 m to approximately −106 dBm at 600 m. This RSSI trend is the measurement most directly linked to the received power and log-distance path-loss terms in Equations (1) and (2). To provide a quantitative deployment-planning reference, the available RSSI scatter data were fitted to the log-distance path-loss model in Equation (2) using least-squares regression. The fit used $d_{0}=1$ m and path-loss values computed as $PL=P_{t}+G_{t}+G_{r}-\mathrm{RSSI}$, with $P_{t}=20$ dBm and combined antenna gain $G_{t}+G_{r}=4$ dBi. The resulting parameters were n=3.50 with a 95% CI of [2.48, 4.52], $PL(d_{0})=27.2$ dB, $\sigma_{\mathrm{sh}}=4.47$ dB, and $R^{2}=0.9022$. Because the measurement route combined open-field LoS segments with partially obstructed NLoS segments, and LoS/NLoS labels were not recorded for each point, these fitted values should be interpreted as aggregate field descriptors rather than separate LoS and NLoS channel models. The measured RSSI values remained above the typical receiver sensitivity threshold for SF7 (approximately −123 dBm), with the weakest measured point (−106 dBm at 600 m) still providing an approximate 17 dB link margin. SNR values remained positive up to approximately 300 m and decreased to approximately −5 to −7 dB at 400–600 m. Despite reduced SNR at extended distances, successful packet reception was maintained within the tested hop range, and all measured SNR values remained above the SF7 demodulation threshold of approximately −7.5 dB. Because the measured LoRa tier is airtime- and protocol-limited, the Shannon expression in Equation (4) is treated only as an upper envelope rather than as a predictive throughput model.

## 9. Discussion

The experimental results demonstrate that the proposed hierarchical architecture achieves reliable operation under realistic agricultural conditions while preserving deployment autonomy. The LoRaMESH tier maintained packet delivery ratios above 90% across up to three hops, confirming that controlled flooding combined with Listen-Before-Talk (LBT) provides sufficient reachability in sparse, obstacle-prone environments. Relative to conventional star-topology LPWAN designs, the multi-hop arrangement is expected to provide greater placement flexibility when direct gateway communication is obstructed by terrain or vegetation. However, because a side-by-side LoRaWAN benchmark was not performed on the same testbed, this comparison should be interpreted as architectural reasoning rather than direct experimental evidence of performance superiority.

The measured end-to-end latency values, ranging between 10 and 13 s on average, reflect the inherent trade-off between long-range communication reliability and airtime efficiency in LoRa communication. While such delay is acceptable for periodic environmental monitoring tasks (e.g., soil moisture, temperature, and crop-state sensing), it may not be suitable for latency-sensitive control applications. In these cases, adaptive duty cycling, selective forwarding, or hybrid routing mechanisms could reduce forwarding delay and channel occupancy without compromising reliability.

At the aggregation layer, the Wi-Fi HaLow backhaul achieved near-perfect reliability at short-to-medium distances, supporting its feasibility as a private sub-GHz backbone for the tested rural IoT deployment. The HaLow-based implementation also avoids cellular subscription dependency in this prototype. However, because no NB-IoT baseline was deployed under identical field conditions, the paper does not claim direct experimental superiority over NB-IoT. The observed PDR degradation from 200 m onward highlights the importance of gateway placement, antenna elevation, and terrain-aware deployment planning. These findings indicate that while the hierarchical separation between sensing (LoRaMESH) and aggregation (HaLow) enhances flexibility, careful physical-layer design remains essential for maintaining stable backhaul links.

Beyond the measured field results, scalability is governed primarily by channel occupancy within the LoRaMESH tier. Under the tested configuration ($T_{s}=10$ s, 64-byte payload, SF7, 125 kHz), the normalized channel load calculated according to Equations (8) and (16) is G≈0.015 N. Maintaining operation in a non-congested regime (G<0.5) therefore suggests a practical cluster size below approximately 30–35 active nodes for the present flooding configuration. As node density increases, LBT-induced deferral and residual collisions caused by hidden terminals become more likely, potentially reducing PDR and increasing latency according to Equations (20) and (21). This analytical projection suggests that controlled flooding is effective for sparsely distributed agricultural sensing but may require adaptive relay selection or cluster segmentation for denser deployments.

Using the explicit airtime substitution in Equation (15) and the representative planning value $k_{f}\approx 1.3$ in Equation (16), this paper now treats the 30–35 node range as an analytical projection rather than as a validated hard limit. The sensitivity analysis in Table 3 shows that this range shifts materially with forwarding redundancy, reinforcing that denser deployments require either direct validation or simulation support before stronger claims can be made.

Overall, the hierarchical architecture provides a practical balance among coverage, reliability, throughput, and infrastructure independence in the tested deployment. The combination of multi-hop LoRa sensing with a private Wi-Fi HaLow backhaul appears suitable for large, sparsely distributed agricultural environments where autonomy is prioritized over ultra-low latency. At the same time, the analytical scalability projection clarifies practical deployment limits and provides guidance for density-aware network planning in future large-scale implementations. These conclusions remain specific to the evaluated architecture and field conditions until direct LoRaWAN or NB-IoT baseline measurements are available.

Using the corrected HaLow dataset, only the 50 m, 100 m, 200 m, and 300 m backhaul distances were measured. The 200 m point already dropped to 89.68% PDR, so the practical high-reliability region is shorter than previously implied, even though the link remained operational at 300 m.

## 10. Deployment Considerations

Practical deployment of the proposed architecture requires consideration of gateway density, antenna placement, and power provisioning. Based on the experimental results, a LoRaMESH hop distance of approximately 400–500 m provides stable communication under mixed LoS/NLoS conditions. Therefore, cluster planning should ensure inter-node spacing within this range to maintain high reliability. For the HaLow backhaul, high reliability was observed up to approximately 100 m, while 200 m remained operational with reduced PDR under the tested conditions. Gateway elevation and directional antennas can extend this range. In large farms, multiple HaLow access points may be required to ensure consistent backhaul performance.

Energy consumption measurements indicate an average end-node sleep current of 21.19μ A and a transmission current of approximately 185mA during a 3.43s transmission interval. Assuming a 2400 mAh lithium battery, the estimated end-node operational lifetime strongly depends on the sensing interval. For a short reporting period of 2 min, the expected lifetime is approximately 19 days. When the sensing interval is extended to 30 min, the estimated lifetime increases to approximately 268 days (about 8.8 months). These results confirm that transmission energy dominates the end-node power budget, and that 1-year autonomous end-node operation would require either substantially longer reporting intervals, transmission time optimization, or energy-harvesting support. This estimation neglects battery self-discharge and environmental degradation.

The new lifetime mapping in Table 5 makes the dependence on sensing interval explicit and should be interpreted only for end nodes. Table 6 clarifies the role-level power assumptions: relay nodes and dual-radio gateways are externally powered in the current design, so no relay/gateway battery lifetime is claimed. Their receive, channel-sensing, forwarding, and Wi-Fi HaLow backhaul current profiles remain relevant for power-supply sizing and are left for future profiling work.

From an infrastructure perspective, the architecture enables private network deployment without reliance on licensed cellular operators, which may reduce recurring operational costs in rural settings. This cost-related interpretation is qualitative because no same-testbed NB-IoT or LTE deployment was included in the experimental campaign.

No 250 m HaLow measurement was collected in the final dataset. Accordingly, the practical interpretation of the current measurements is that the backhaul remains highly reliable at short distances, while 200 m should be treated as a degraded but still operational regime under the tested field conditions.

## 11. Limitations

Despite the positive experimental results, several limitations should be acknowledged. First, the LoRaMESH routing mechanism is based on controlled flooding, which may introduce redundant retransmissions and increased collision probability in dense network deployments. The present evaluation focuses on sparse agricultural scenarios; scalability under high node density requires further investigation, and the current 30–35 node figure should be read as an analytical planning result rather than as a fully validated deployment limit. No denser field deployment or simulation sweep was performed to validate the analytical scalability limit under varying node densities, and the collision model should be interpreted as a first-order approximation. No node-failure, link-failure, or rerouting-recovery experiment was conducted; therefore, path-redundancy or fault-tolerance should be an expected design property of the controlled-flooding mesh architecture rather than as an experimentally demonstrated resilience outcome.

Second, the Wi-Fi HaLow evaluation was conducted within a maximum tested distance of 300 m under specific environmental conditions. Performance in highly obstructed or mountainous terrain may differ and warrants additional study.

Third, latency measurements were obtained under a fixed packet interval of 10 s. Different traffic patterns, such as burst transmissions or event-driven sensing, may influence delay characteristics and network stability.

Finally, and most importantly for comparative interpretation, the experiments did not include a direct side-by-side comparison with LoRaWAN or NB-IoT deployments under identical field conditions. While qualitative comparisons are discussed, quantitative benchmarking against alternative LPWAN systems remains future work. Therefore, the reported results validate the feasibility of the proposed architecture under the tested conditions but do not establish direct performance superiority over LoRaWAN, NB-IoT, or other LPWAN baselines.

Three additional limitations are important for interpretation. The analytical expressions for airtime, throughput, normalized load, and LBT success probability are approximate design tools, not fully validated predictive models, because $k_{f}$, $P_{\mathrm{busy}}$, and residual hidden-terminal effects were not independently measured at the packet level. The propagation measurements combine mixed LoS and NLoS conditions, and packet-level logs with per-sample LoS/NLoS labels were not preserved. Therefore, the log-distance fit reported in Figure 7 should be treated as an aggregate deployment-planning descriptor, not as a statistically generalizable or class-specific channel model. Moreover, the security treatment is partial: application-layer AES-CTR payload encryption is mentioned, but replay resistance, and gateway/backhaul hardening were not experimentally validated in this study.

## 12. Conclusions

This work presented a hierarchical wireless communication architecture integrating LoRaMESH multi-hop sensing with a Wi-Fi HaLow backhaul for large-scale smart agriculture. One of the first real outdoor agricultural validations of this design space was conducted through a field deployment covering up to 2.5km×1km to evaluate reliability, latency, and energy performance.

Experimental results demonstrate that the LoRaMESH network maintains over 90% PDR across three hops under mixed propagation conditions. The Wi-Fi HaLow backhaul achieved near-perfect reliability at short distances and remained operational up to 300 m, with clear degradation from 200 m onward. Energy measurements quantified the battery-powered end-node sleep and transmission profile, while the battery-life analysis indicates that 1-year end-node operation would require longer reporting intervals or energy-harvesting support. Relay nodes and gateway nodes are externally powered in the current design, so no whole-network battery lifetime is claimed.

The findings validate the feasibility of combining multi-hop LoRa sensing with sub-GHz IP-based aggregation under the tested agricultural field conditions. They do not establish direct performance superiority over LoRaWAN or NB-IoT because no same-testbed baseline benchmark was conducted. Future work will follow a concrete validation roadmap: implementing a proper routing protocol such as RPL or AODV adapted for low-rate LoRa links; testing higher spreading factors for extended-range operation; integrating real agricultural sensors instead of synthetic packet generators; conducting denser scalability experiments and simulation sweeps under varying node densities; collecting packet-level propagation logs for separate LoS/NLoS channel fitting; profiling relay and gateway power consumption for external-power sizing; running a direct LoRaWAN or NB-IoT field baseline; and strengthening the end-to-end security framework with documented key management and authenticated integrity protection.

## Figures and Tables

**Table 1:** Comparison with representative published baselines.

Reference	Technology/ Topology	Domain	Validation	Reported Scale/Range	Reported Performance	Main Gap Relative to This Work
Codeluppi et al. [26]	LoRaWAN star with multi-protocol gateway	Smart farming	Real field	3 ha farm; 20 × 9 × 5 m greenhouse	PDR about 81–83% for three of four nodes and 70% for one greenhouse node at SF7	No LoRa mesh tier or Wi-Fi HaLow/IP backhaul hierarchy
Rivera-Guzman et al. [23]	LoRa with local Wi-Fi gateway; mainly single-hop	Agriculture	Real field	50 ha; up to 875 m	RSSI down to −122 dBm; PDR 76–92%	No Wi-Fi HaLow; no multi-hop LoRaMESH backhaul integration
Truong [24]	LoRa–Zigbee hybrid WSN	Heterogeneous WSN	Prototype evaluation	Zigbee 630 m; LoRa 3.7 km	Packet loss <0.5%	Not focused on agricultural sensor-to-cloud backhaul
Cotrim et al. [12]	Multi-hop LoRa underground line network	Agriculture	Real field	Buried linear topology; hourly reporting	100% delivery; round-trip delay <200 s	Underground linear deployment; no IP backhaul tier
Alhomyani et al. [13]	Multi-hop LoRa linear network	Pipeline monitoring	Analytical model	Large linear infrastructure	Throughput-energy tradeoff study	No field deployment; no Wi-Fi HaLow tier
Durand and Booysen [14]	LoRa mesh LPWAN	General IoT	Simulation	Dense network; nodes beyond 5.8 km	PDR improved from 40.2% to 73.78% at long range	Simulation only; not agricultural or dual-radio
Kane et al. [15]	Wi-Fi HaLow versus LoRa	Smart grid	Real field	Outdoor field comparison	HaLow achieved higher throughput and lower latency than LoRa	Compares technologies, but does not integrate them in one architecture
Maudet et al. [19]	Wi-Fi HaLow point-to-point	General IoT	Practical measurements	Up to 1 km at 23 dBm	Nearly 6 Mbps at 2 MHz; practical propagation model	No LoRa sensing tier or agricultural field architecture
This work	LoRaMESH + Wi-Fi HaLow dual-radio hierarchy	Agriculture	Real field	Up to 2.5km×1km ; HaLow tested to 300 m	LoRaMESH PDR >90% across 1–3 hops; HaLow PDR 99.5% at 50 m and 89.68% at 200 m	Adds measured agricultural sensor-to-cloud validation, but still lacks direct LoRaWAN/NB-IoT side-by-side benchmarking

**Table 3:** Sensitivity of the normalized-load coefficient to the forwarding factor $k_{f}$ for $T_{s}=10$ s and $T_{\mathrm{air}}\approx 118$ ms.

$k_{f}$	Approximate Load Model	$N_{\max}$ for G<0.5
1.0	G≈0.0118N	42
1.3	G≈0.0153N	32
1.5	G≈0.0177N	28
2.0	G≈0.0236N	21

**Table 4:** Delay statistics for different hop scenarios.

Hop Count	Mean (s)	Std (s)	Min–Max (s)
1-hop	10.62	5.13	4.62–33.56
2-hop	10.81	4.93	2.31–23.14
3-hop	13.31	6.72	3.10–43.64

**Table 5:** Estimated end-node lifetime for a 2400 mAh LiPo battery under different sensing intervals.

Sensing Interval	Average Current	Estimated Lifetime
2 min	5.320 mA	18.8 days
5 min	2.141 mA	46.7 days
10 min	1.081 mA	92.5 days
20 min	0.551 mA	181.5 days
30 min	0.374 mA	267.1 days
60 min	0.198 mA	505.5 days

**Table 6:** Power-source assumptions and energy-analysis scope by node role.

Node Role	Power Source in This Design	Energy-Analysis Scope
End node	Battery-powered	Sleep and transmission current were measured using PPK2; battery lifetime was estimated for different sensing intervals.
Relay node	Continuous external power	Battery lifetime was not estimated because relay nodes are not battery-powered in the current design. Receive, LBT/channel-sensing, and forwarding current remain relevant for power-supply sizing but were not separately instrumented.
Gateway node	Continuous external power	Battery lifetime was not estimated because gateway nodes are externally powered and include both LoRa and Wi-Fi HaLow radios. Gateway LoRa reception, processing, bridging, and HaLow backhaul current remain future profiling work.

## Data Availability

The data presented in this study are available on request from the corresponding author. The data are not publicly available due to experimental dataset size and field deployment constraints.

## Abbreviations

**Abbreviation**	**Meaning**
WSN	Wireless Sensor Network
IoT	Internet of Things
LPWAN	Low-Power Wide-Area Network
QoS	Quality of Service
PDR	Packet Delivery Ratio
LoS	Line-of-Sight
NLoS	Non-Line-of-Sight
AP	Access Point
STA	Station
TTL	Time-To-Live
LBT	Listen-Before-Talk
LiPo	Lithium-polymer battery
RSSI	Received Signal Strength Indicator
SNR	Signal-to-Noise Ratio
SINR	Signal-to-Interference-plus-Noise Ratio
MCU	Microcontroller Unit
PHY	Physical Layer
MAC	Medium Access Control
SDN	Software-Defined Networking
NB-IoT	Narrowband Internet of Things
LoRaWAN	Long Range Wide Area Network
LoRaMESH	LoRa Mesh (multi-hop LoRa network)
IEEE	Institute of Electrical and Electronics Engineers
SF / SF7	Spreading Factor (7)
BW	Bandwidth
CR	Coding Rate
CRC	Cyclic Redundancy Check
AES	Advanced Encryption Standard
SPI	Serial Peripheral Interface
ADC	Analog-to-Digital Converter
I2C	Inter-Integrated Circuit
DC-DC	Direct Current to Direct Current (converter)
VDD	Supply voltage
PPK2	Power Profiler Kit II (Nordic)
MCS	Modulation and Coding Scheme
RAW	Restricted Access Window
TWT	Target Wake Time
CI	Confidence Interval
CC BY	Creative Commons Attribution
3GPP	3rd-Generation Partnership Project
ITU-R	International Telecommunication Union—Radiocommunication
LTE	Long-Term Evolution
